# Longitudinal Testing of Exploratory Behaviour in Mice Reveals Stable Cognitive Traits Across the Adult Lifespan

**DOI:** 10.1111/acel.70287

**Published:** 2025-11-13

**Authors:** R. Abuhamdah, G. Moore, D. Djama, F. Zirpel, C. Edge, A. Ennaceur, P. Chazot, D. Cash, E. Kim, A. C. Vernon, P. Chadderton, S. G. Brickley

**Affiliations:** ^1^ Imperial College London London UK; ^2^ University of Sunderland Sunderland UK; ^3^ Durham University Durham UK; ^4^ Kings College London London UK; ^5^ University of Bristol Bristol UK

## Abstract

In human populations, cognitive performance in early life has been identified as a strong predictor of future dementia risk. If cognitive ability were a stable trait across the adult lifespan of mice, then this species would provide an excellent framework for understanding the biology underlying this modifiable risk factor. To address this issue, longitudinal cognitive testing was performed in female C57BL/6J mice aged between 4 and 18 months of age. By tracking individuals, we were able to demonstrate that the cognitive performance of an animal in specific tasks at 4 months of age was a remarkably reliable indicator of performance at 18 months of age. Variability in the performance of individuals was not associated with differences in macroscopic brain structure, but single‐cell recording from neurons of the prefrontal cortex did identify age‐related changes in membrane excitability. Most importantly, this study demonstrated that strategies adopted early in life to explore a nine‐arm radial maze were maintained across the adult lifespan and learning effects associated with repeated exposure to a test contribute to this stability. Overall, our results demonstrate that, like humans, cognitive ability in mice is a stable trait and the cognitive reserve necessary for healthy brain ageing is established early in life.

## Graphical abstract

Video tracking of mice demonstrated how exploratory performance at 4 months of age was a strong predictor of performance at 18 months of age. This longitudinal testing revealed that cognitive ability is a stable trait in adult mice that was associated with stable brain morphology and neuronal excitability.

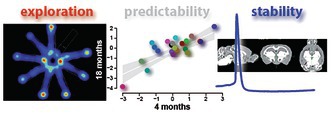

## 1 Introduction

Longevity is a defining accomplishment of modern societies, but human ageing is too often accompanied by morbidities that result in loss of functional independence (Wittenberg et al. 2019). In the absence of effective treatments, studies have attempted to track the trajectory of individuals across the lifespan to identify modifiable risk factors for sporadic forms of dementia. Longitudinal testing in humans has identified multiple factors that could enable cognitive performance to be maintained into later life (Lipnicki et al. 2019) and ageing studies across species have revealed biomarkers associated with the ageing process that could be exploited to reduce dementia rates (Perlman 2016; Brito et al. 2023; Weber et al. 2015; Prince et al. 2013). Arguably, one of the most striking observations of longitudinal studies in human populations is that cognitive performance in early life is a strong predictor of performance in later life. The relationship between cognitive performance in early life and reduced dementia risk in later life (Livingston et al. 2024) highlights the need to understand how the cognitive reserve that underlies resilience is established. Few studies have, to our knowledge, examined whether cognitive abilities in mice are stable across the adult lifespan in an analogous manner to humans. In this study, we have undertaken longitudinal cognitive testing in group‐housed female C57Bl/6J mice paying particular attention to how the performance of individual mice alters over the testing period. Cognitive testing began at 4 months of postnatal age and was repeated regularly until the mice were 18 months of age. This upper limit was chosen to avoid the complication of clinical frailty (Whitehead et al. 2014). Open field tests were used to monitor chronic stress levels (Katz et al. 1981) and object location/recognition tests were carried out to identify changes in short‐term memory. A nine‐arm radial maze test (Abuhamdah et al. 2016) was chosen to quantify age‐related changes in long‐term memory, as well as giving some insight into decision‐making and exploratory behaviour with a particular emphasis on learning strategies. Correlation analysis of data obtained between 4 and 18 months of age was used to test our hypothesis that performance in early life would be a strong predictor of cognitive performance at older ages. At the beginning and end of behavioural testing, whole‐cell recording was undertaken to quantify possible differences in neuronal excitability, concentrating on neurons of the prefrontal cortex where age‐related changes have often been reported (Lucaci et al. 2023; Moore et al. 2023). At completion of longitudinal testing, whole brain imaging was used to identify any associations between cognitive performance and cortical/subcortical brain volumes. The main aim of this work was to determine if cognitive abilities in early life could be used to predict outcomes in later life to establish a useful model for the study of healthy brain ageing.

## 2 Methods

### 2.1 Animals

During this study, a total of 58 C57BL/6J (Charles River, UK) female mice were housed 4 per cage under a 12‐h light: dark cycle (light 07:00–19:00 h) at 23°C ± 1°C with ad libitum access to food and water. Animal treatment and husbandry were carried out in accordance with the Animals (Scientific Procedures) Act 1986, UK. Consistent with previous Kaplan–Meier survival analysis for female C57BL/6J mice, three animals from this cohort were euthanised during this study because of apparent poor health and 14 animals were euthanised for electrophysiological experiments (see below).

### 2.2 Electrophysiology

**FIGURE 1:**
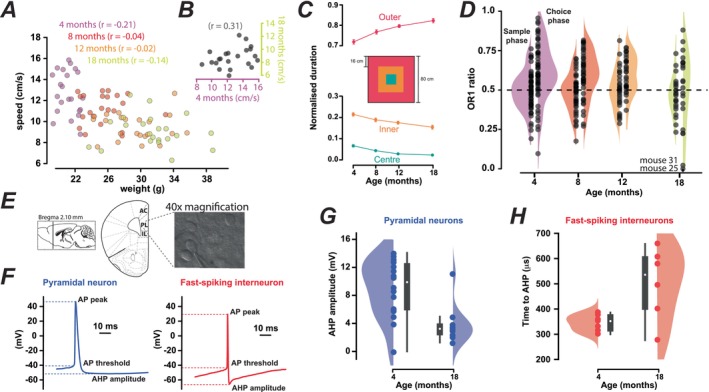
Age‐related changes in body weight, distance moved, and apparent anxiety levels were detected in a cohort of female mice in which age‐related changes in AP properties are also observed. (A) Scatter plots showing the relationship between the animals' body weight and their exploratory speed at each of the age ranges examined. A Welch's one‐way ANOVA with Dunnett's correction for post hoc multiple comparisons demonstrated a significant increase in body weight between 4 and 8 months of age (*p* < 0.0001), between 8 and 12 months of age (*p* < 0.0001), and between 12 and 18 months of age (*p* < 0.0001). Analysis of video tracking data from the open‐field tests identified an age‐related reduction in exploratory time in the open field arena with a mean speed of 12.71 ± 0.44 cm/s (*n* = 22) at 4 months, 11.19 ± 0.30 cm/s (*n* = 22) at 8 months, 9.48 ± 0.27 cm/s (*n* = 22) at 12 months and 9.08 ± 0.32 cm/s (*n* = 22) at 18 months. Welch's one‐way ANOVA with Dunnett's correction for post hoc multiple comparisons demonstrated significant changes between 4 and 8 months of age (*p* = 0.0423), and between 8 and 12 months of age (*p* = 0.0009), but there was no significant difference in the exploratory speed between 12 and 18 months of age. (B) Scatter plot demonstrating the lack of any relationship between the exploratory speed of individual animals at 4 and 18 months of age. (C) Duration of time spent in the centre (blue), inner (orange) and outer (red) regions of the arena (shown) normalised to total exploration time (mean ± SEM). With age, mice showed increased exploration of the outer region adjacent to the wall with a reduction of time spent in the centre or inner regions. (D) Plots of data from novel object recognition and object location tests comparing the exploration ratios following 1‐ and 15‐min retention times for mice at 4, 8, 12 and 18 months of age. During this task, a total of 58 female C57Bl/6J mice were tested at 4 months of age, resulting in a mean preference ratio (OR1) of 0.59 ± 0.05 in the choice phase of the trial compared to a ratio of 0.49 ± 0.01 during the preceding sample phase when the animal's exploration of the familiar object was close to chance levels. A total of 46 animals were tested at 8 months, with an elevated OR1 of 0.60 ± 0.02 in the choice phase of the trial compared to 0.48 ± 0.02 in the sample phase. At 12 months of age, 31 mice were tested, with an elevated OR1 of 0.60 ± 0.02 in the choice phase of the trial compared to 0.49 ± 0.02 in the sample phase. Finally, at 18 months of age, results from 21 mice gave an OR1 of 0.46 ± 0.02 in the choice phase of the trial that was not statistically different from the OR1 of 0.49 ± 0.02 calculated in the sample phase (Welch's one‐way ANOVA). However, when mouse 25 was removed, the OR1 score at 18 months of age was still low at 0.53 ± 0.05 (*n* = 20) but, like the other ages examined, this was significantly different from the OR1 calculated in the sample phase of the test at 0.46 ± 0.02 (*n* = 20). (E) Images from the Allan Brain Atlas illustrate the recording location used to make whole‐cell current clamp recordings from pyramidal neurons and fast‐spiking interneurons of the PFC. A 40‐image from the fixed‐state microscope has been included to clarify our visually guided cell identification procedure. (F) A representative example of an action potential (AP) recorded from a pyramidal neuron (blue trace) and a fast‐spiking interneuron (red trace) with dashed lines indicating the location of the AP threshold, AP peak, and the hyperpolarisation (AHP) phase of the AP. The bottom plot illustrates the clear difference in maximum AP firing rates calculated in these two neuronal types following steady‐state current injection protocols (examples not shown). (G) Scatter and violin plots of the age‐related change in the AHP magnitude for pyramidal neurons (blue circles). (H) Scatter and violin plots of the age‐related change in the time to AHP for fast‐spiking interneurons (red circles).

Recordings were made in the prefrontal cortex (PFC) from 7 mice aged 4 months postnatal and 7 mice at 18 months postnatal as described previously (Lucaci et al. 2023). In brief, acute slice preparations were prepared at a thickness of 250 μm, after which neurons were visualised with a high numerical aperture water‐immersion objective connected to an infrared‐sensitive digital camera. Whole‐cell recordings were made from fast‐spiking interneurons (FS‐INs) and pyramidal neurons (PyrNs) within layer 2/3 of the prelimbic (PL), anterior cingulate (AC), and infralimbic (IL) regions of the PFC (see Figure 1E). Recordings were made using patch pipettes with a resistance of 3–4 MΩ when back‐filled with internal solution that contained (in mM): 145 K‐gluconate, 4 NaCl, 0.5 CaCl_2_, 10 HEPES, 5 EGTA, 4 Mg‐ATP, and 0.3 Na‐GTP, adjusted to pH 7.3 with KOH. The extracellular recording solution contained (in mM): NaCl 125, KCl 2.5, CaCl_2_ 2, MgCl_2_ 1, NaH_2_PO_4_ 1.25, NaHCO_3_ 26, and glucose 11, at pH 7.4 when bubbled with 95% O_2_/5% CO_2_. The amplifier head stage was connected to an Axopatch 700B amplifier (Molecular Devices), and the voltage output was filtered at 10 kHz (−3 dB, eight‐pole low‐pass Bessel filter) and digitised at 20 kHz using a CED Power 1401 processor running CED Signal (version 6) software. PyrNs and FS‐INs were selected according to their location, soma shape, and electrophysiological features such as membrane capacitance (*C*_m_ = *Q*/Δ*V*, where *Q* is the charge transfer during a change in the command voltage, Δ*V*), and membrane conductance (*G*_m_ = *I*_ss_/Δ*V*, where *I*_ss_ is the average steady‐state current).

### 2.3 Nine‐Arm Radial Maze

All behavioural experiments were recorded (800 × 600 pixels, 14.8 frames per second) and movements tracked using EthoVision software (Noldus Information Technology, USA). The radial maze (RM) consisted of a central platform connected to each of the nine arms by bridges at a 40° incline (see Figure 1A). As described previously (Abuhamdah et al. 2016), a unique visual cue was positioned at the end of each arm that could only be observed once the mouse had fully climbed the bridge. A food pellet was placed at the end of each arm beneath the visual cue. During each testing period, mice were subjected to 16 days of RM testing. One day prior to RM testing, mice were individually weighed and food‐restricted. Weight monitoring was continued throughout the testing stage to ensure mice did not drop below 90% of their baseline, in accordance with animal welfare protocol regulations. On each test day, mice were introduced into the central platform of the maze and left to explore the RM, where food was available at the end of each arm. The task was concluded after 10 min of exploration time or once mice had explored any nine arms. To prevent mice from associating an arm location with task completion we allowed mice to return to the centre of the maze before removal from the arena. The maze was disinfected between each trial to remove odour cues.

### 2.4 Open‐Field and Novel Object Location and Recognition

All mice were also placed in an open‐field arena that was also used for object location and recognition testing (see Figure 1C). The open‐field, novel object recognition and location tasks were performed prior to the 16‐day radial maze session. The order of testing for mice was 3 days in the open‐field, 4 days in object recognition and location, and 16 days in the radial maze, amounting to 23 days of testing overall at each age point. During open‐field testing, mice were placed into the central area and allowed to explore the empty arena for a total of 10 min on 3 consecutive days. Mice were removed and the arena sterilised with disinfectant between each experiment. For novel object location and recognition testing, we placed novel and familiar objects at any of the four vertices adjoining the inner and outer areas. They were therefore located 16 cm from the arena wall. During testing, mice were placed in the central area and given 3 min to explore two identical objects placed at two of the four possible object locations. Mice were removed for a 1‐ or 15‐min retention interval. For object location testing, one of the objects was moved to a new location. For object recognition testing, one of the objects was replaced with a differently shaped novel object. Mice were returned to the arena after the retention interval and left to explore for a further 3 min. Following task completion, mice were removed and returned to their cages, and the open‐field arena was cleaned with disinfectant. On successive days, mice were returned to the arena to complete all variants of the task such that each mouse was subject to 1‐ and 15‐min retention intervals for object recognition (OR1 and OR15) and object location tasks (OL1 and OL15).

### 2.5 Radial Maze, Objection Location and Object Recognition Performance Analysis

Performance measures were extracted from video tracking data.

#### 2.5.1 Decision‐Making Time (DMT)

Mean decision‐making time, $\bar{\mathrm{DMT}}$, was calculated according to the following.

(1) $$\bar{\mathrm{DMT}}=\frac{T_{\text{trial}}}{16\left(n+1\right)}$$

$T_{\text{trial}}$ represents the time spent in the trial (either 10 min or less if nine arms were visited), n represents the number of arms visited, incremented by +1 so a baseline of zero arm visits results in a real $\bar{\mathrm{DMT}}$ value, and 16 represents the number of trial days.

#### 2.5.2 Number of Errors (E)

Mean error, $\bar{E}$, was calculated with the following.

(2) $$\bar{E}=\frac{\sum E}{16}$$

E represents the errors on each of the 16 trial days. An error is recorded when a mouse returns to a previously visited arm in a session.

#### 2.5.3 Unique Sequence Score (USS)

All unique sequence lengths (1–9) prior to an error (revisiting an already explored arm in that sequence) were identified during each trial. Identification of a unique sequence was not limited to the start of the trial, and each unique sequence could not belong to another unique sequence. The USS was then calculated from the weighted Euclidean sum square product score,

(3) $$\mathrm{USS}=\sqrt{\sum_{i=1}^{9}{\left(if_{i}\right)}^{2}}$$

f represents the frequency of sequence length i. As the sequence length weighted the score, mice with few long‐length unique sequences and fewer unique sequences overall, achieved a higher *Z*‐score than mice with a higher frequency of low‐length unique sequences.

#### 2.5.4 Learning Rate

Linear regression was used to determine the rate of change of error for each mouse across the 16‐day testing session. The learning rate was calculated by multiplying the error rate by −1, such that high errors early in the test session with low errors later in the test session corresponded with high learning ability. This meant that high‐learning mice reduced the errors they accumulated over the RM testing period. A high *Z*‐score corresponded to a higher learning rate.

#### 2.5.5 Exploration Ratio

The radial maze was split into 28 regions: 9 arms, 9 bridges, 9 outer‐centre and 1 inner‐centre. Video tracking was used to measure how many regions were explored by each mouse. The mean exploration amount was calculated as the sum of all explored regions divided by the total possible number. As mice were introduced to the maze using the inner‐centre area during the trial, this region was omitted from the analysis, giving a total of 27 explorable regions per day.

#### 2.5.6 Cumulative Arm Time

This was the mean total time each mouse spent on the arms, with a higher cumulative arm time giving a higher *Z*‐score.

#### 2.5.7 Speed

Video tracking data were used to measure the total distance travelled by each mouse in the nine‐arm radial maze, and this was divided by the time spent in the trial to estimate the average speed across the 16 trial days. Mice with greater velocities were, therefore, ranked with a higher *Z*‐score.

#### 2.5.8 Preference Ratio

Following a 1‐ or 15‐min retention interval between the sample and choice phase, the preference ratio was calculated with the following formula for recognition and location tests.

(4) $$\text{Preference}=\frac{T_{\text{novel}}}{T_{\text{novel}}+T_{\text{familiar}}}$$

$T_{\text{novel}}$ represents the time spent with the novel object during the choice phase, and $T_{\text{familiar}}$ represents the time spent with the familiar object during the choice phase.

### 2.6 Combining Different Measures of Performance

To directly compare inter‐ and intra‐animal performance at different ages, we calculated the *Z*‐score for each metric, allowing different aspects of cognitive performance to be compared across the ages examined (Walker et al. 2018; Guilloux et al. 2011). The open‐field measures were not included in subsequent analysis as the task was used to assay anxiety levels and was not used to assay cognitive performance. For each metric, an animal's *Z*‐score was calculated as the animal's performance, x, minus the population mean, μ, divided by the standard deviation, σ.

(5) $$Z_{i}=\frac{x-\mu }{\sigma }$$

Individual animals that performed exceptionally well or poorly in the metric had a more positive or negative *Z*‐score. The *Z*‐scores obtained for $\bar{\mathrm{DMT}}$ and $\bar{E}$, were inverted to correlate a higher *Z*‐score value with better performance, and all *Z*‐scores were then combined into a single representative *Z*‐score using the following Stouffer's method.

(6) $$Z=\frac{\sum_{i}^{k}Z_{i}}{\sqrt{k}}$$

$Z_{i}$ represents the *Z*‐score for metrics i, and k represent the number of metrics. The open‐field measures were not included in subsequent analysis as the task was used to assay anxiety levels and was not used to assay cognitive performance.

#### 2.6.1 Weighted Linear Regression Analysis

To avoid over‐fitting of longitudinal data, a regularisation approach was implemented. The additional error function (J) incorporated into the least‐squares fitting routine was.

(7) $$J=\sum_{i=1}^{n}{\left(y_{1}-\left(w_{0}+w_{0}x_{1}\right)\right)}^{2}$$

where n is the sample size, $w_{0}$ is the gradient and $w_{1}$ is the intercept of the fitted line for each $y_{1}$.

#### 2.6.2 Pearson's Correlation Coefficient

Following the least‐squares fitting of data with the weighted linear function, covariance between variables was determined with Pearson's correlation coefficient (r) that was calculated according to.

(8) $$r=\frac{\sum_{i=1}^{n}\left(x_{i}-\bar{x}\right)\left(y_{i}-\bar{y}\right)}{\sqrt{\sum_{i=1}^{n}{\left(x_{i}-\bar{x}\right)}^{2}\;}\;\sqrt{\sum_{i=1}^{n}{\left(y_{i}-\bar{y}\right)}^{2}\;}}$$

where n is the sample size, $x_{i}$, $y_{i}$ are indexed values, and $\bar{x}$, $\bar{y}$ are the sample means for each variable.

### 2.7 Magnetic Resonance Imaging

A subset of mice (14 from the final 22) that completed all radial maze, novel object location and novel object recognition tests was allocated to ex vivo structural MRI. These data were acquired using a 9.4 Tesla pre‐clinical MR system (Agilent Technologies, USA). Mice were culled by IP injection of sodium pentobarbital (50 mg/kg) followed by transcardial perfusion with 20 mL of 1× PBS (pH 7.4) at 4°C, followed by 20 mL of 4% PFA at 4°C. Brain tissue (intact in the cranium) was then stored in 4% PFA at 4°C for 24 h before washing three times with 1× PBS and storing in 1× PBS with 0.01% NaN_3_ until MR imaging. Four samples were imaged at a time by positioning them inside a Falcon tube and separating them with a polypropylene divider. Samples were immersed in an inert fluorinated solution (Fomblin, Solvay), and the following images were acquired: T1‐weighted (MP2RAGE), T2‐weighted (FSE) and diffusion (DTI) scan configuration, with isotropic voxels (1 mm^3^). MR images were converted to the NIFTI format and analysed using FMRIB Software Library (FSL; Jenkinson et al. 2012). For (total) grey matter and white matter segmentation, FMRIB's Automated Segmentation Tool (FAST) was used on T1‐weighted MR images (Zhang et al. 2001). FAST has the benefit of being more robust and less sensitive to noise and implements bias field correction to reduce low‐frequency bias in the magnetic field.

### 2.8 Image Processing and Analysis

Images were resized in ImageJ to fit the dimensions of the registration template available at the Allen Brain Institute. This allowed images to be resized from 125 × 191 × 96 and 1 mm^3^ voxel size to 228 × 264 × 160 with a voxel size of 0.1 mm^3^. A median filter of 0.1 mm^3^ was also applied to the images to reduce noise. The BET tool was used to remove image artefacts surrounding the brain tissue. Subsequently, the SUSAN function was used to reduce noise with a Mask Standard Deviation of ‘0’ for faster smoothing as recommended. Registration was conducted using the FLIRT function using the brain template available at the Allen Brain Institute. An Affine 12‐parameter model was used to increase the dimensions of registration. Segmentation was performed using the FAST function with a Markov Random Field parameter of 0.1, Bias Field Smoothing of 5.0 mm as well as Bias Field removal. Both partial and non‐partial images of total Grey Matter, White Matter & Cerebrospinal Fluid were obtained, and non‐partial images were used for quantification. Left/right asymmetry was assessed by segmenting the whole brain as well as White and Grey Matter images along the midline and conducting a Student's t‐test to evaluate the differences. Segmentation of the thalamus, hippocampus, prefrontal cortex, striatum and cerebellum was performed using ITK‐Snap's snake segmentation tool once the annotated atlas offered by the Allen Brain Institute was registered to the resized T2‐weighted images using FSL's linear FIRST package. Different cortical and subcortical **v**olumes were calculated using the FSL command line code and compiled in Excel.

### 2.9 Statistical Analysis

All measures obtained at 4 months of age were averaged for each animal (*n* = 58) and tested with the D'Agostino‐Pearson normality test. Welch's one‐way ANOVA was used for hypothesis testing with Dunnett's correction for post hoc multiple comparisons. Consistent with previous behavioural studies (Shoji et al. 2016), the population measures were assumed to be normally distributed but with non‐equal variances across age groups.

## 3 Results

### 3.1 Progressive Changes in Body Weight, Activity and Anxiety Levels in Our Cohort of Female Mice

Adult female mice, that were group housed throughout life, exhibited significant age‐related increases in body weight (tested with one‐way ANOVA with Dunnett's correction for post hoc multiple comparisons) and significant reductions in exploratory behaviour (Figure 1A). Weighted linear regression analysis demonstrated that there was no significant relationship between the weight of the mice and their exploratory behaviour at the ages examined (Figure 1B). Moreover, the speed of the mice recorded at 4 months of age did not correlate with the speed obtained for this cohort at 18 months of age, with a Pearson's *r* = 0.32 and a lack of statistical significance (ANOVA with Dunnett's correction). We did observe age‐related changes in the amount of time spent exploring the centre, inner and outer regions of the open field arena (Figure 1C) with significant changes at all ages (*p* < 0.0001). For example, exploration of the outer region increased significantly (ANOVA with Dunnett's correction) from 72.88% ± 1.09% (*n* = 22) of the total time at 4 months to 82.26% ± 1.05% (*n* = 22) at 18 months at the expense of the proportion of time spent in both the centre and the inner regions of the open field. Our data from the open field tests could imply that anxiety levels are increasing with age, given the animals' reluctance to enter the centre regions of the open field. Speed in the open arena does not appear to be contributing to the increase in time spent in the outer regions, as the relationship between speed and outer preference was not positively correlated (data not shown). Alternatively, it could be argued that an animal's level of arousal or motivation is reducing with age and so age‐related frailty could explain the altered behaviour in the open arena. Importantly, previous studies that quantified skeletal muscle mass and measured parameters such as grip strength support our impression that clinical frailty is not apparent in mice at 18 months of age prior to changes in gene expression in older animals (over 24 months) that were related to loss of skeletal muscle (Kang et al. 2022). Therefore, we are assuming that at 18 months of age, our mice are equivalent to humans of approximately 60 years of age who are entering the early stages of the ageing process with little evidence of clinical frailty.

### 3.2 Short‐Term Working Memory Did Not Decline Over the Period Studied

Age‐related changes in short‐term memory were explored using novel object recognition (OR1 and OR15) and object location (OL1 and OL15) tests, but no age‐related changes were apparent for this cohort following either weighted linear regression analysis or hypothesis testing with ANOVA using Dunnett's correction. We did, however, note that the OR1 score at 18 months included data points from two animals that exhibited very low OR1 ratios of 0 (mouse 25) and 0.02 (mouse 31). These low scores were due to a very low exploration speed for mouse 25 (2.2 cm/s). In contrast, mouse 31 was moving at a normal speed (12.2 cm/s) during this test. Taken together, this analysis demonstrates that there was no statistically significant change in short‐term memory in this cohort of mice between 4 and 18 months of age, even when the impact of inter‐animal variability was reduced with a longitudinal testing approach. This would support the view that group‐housed female C57Bl6 do not exhibit cognitive decline during the normal healthy ageing process, and future studies will be needed to establish if clinical frailty impacts cognitive performance at older ages above 18 months.

### 3.3 Age‐Related Changes in Neuronal Excitability Were Observed in Neurons of the PFC

The excitability of pyramidal neurons and fast‐spiking interneurons in the PFC of female mice (Figure 1E) was compared before 4 months (3.61 ± 0.27 months, *n* = 8 mice) and after 18 months of age (20.55 ± 1.20 months, *n* = 10 mice) at the beginning and end of the longitudinal testing period. We did not observe age‐related changes in cell parameters such as the resting membrane potential and membrane time constant or the AP threshold, AP peak, AP half‐width or maximum AP firing rates recorded from either cell population (Figure 1F). The only parameters that changed significantly with age were related to the afterhyperpolarisation (AHP) of the AP. The peak amplitude of the AHP in pyramidal neurons (Figure 1G) significantly decreased (*p* = 0.0015) from 9.09 ± 1.01 mV (*n* = 16 cells) at 4 months of age to 3.88 ± 0.87 mV (*n* = 10 cells) at 18 months of age, whereas the time to the peak of the AHP (Figure 1H) was significantly delayed (*p* = 0.043) from 345.14 ± 12.02 μs (*n* = 7 cells) at 4 months of age to 502.00 ± 58.44 μs (*n* = 6 cells) at 18 months of age in the fast‐spiking interneuron population.

### 3.4 Exploratory Behaviours Are Maintained From 4 to 18 Months

**FIGURE 2:**
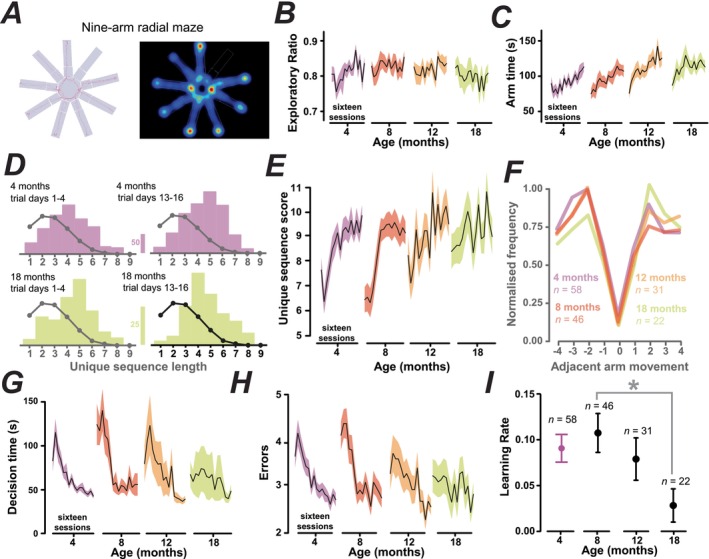
Mice adopt exploratory strategies that are conserved across adult life. (A) Video tracking data for a mouse performing the nine‐arm radial maze task (left, the red line shows animal path) with a corresponding heat map (right). (B) Plots show daily performance (mean ± SEM; black solid and shaded areas) across the 16 sessions for the exploratory ratio. Across the entire cohort, the exploration ratio (27 areas of the maze not including the centre) gave a mean exploration ratio of 0.81 ± 0.01 (*n* = 22) at 4 months, 0.83 ± 0.01 (*n* = 22) at 8 months, 0.82 ± 0.01 (*n* = 22) at 12 months and 0.80 ± 0.02 (*n* = 22) at 18 months. Therefore, this cohort of mice appeared to explore similar areas of the maze at all ages (*p* = 0.46). (C) Plots of cumulative arm time data at different ages. The cumulative arm time did not alter significantly with age (*p* = 0.085) with a mean arm time of 92.38 ± 4.98 s (*n* = 22) at 4 months, 96.39 ± 7.60 s (*n* = 22) at 8 months, 111.80 ± 7.17 s (*n* = 22) at 12 months and 112.70 ± 9.04 s (*n* = 22) at 18 months. (D) Histograms of the frequency of any unique sequence length. Predictions of a stochastic model of the same number of mice are superimposed on each histogram (black line and circles). The stochastic model achieved a modal unique arm sequence value of two. In comparison, across all ages, mice accomplished a modal value of four or five unique arm choices before returning to an arm they had previously visited, incurring an error of one missed arm. Results from testing performed at 4 months [*n* = 58] and 18 month [*n* = 22] are shown in this plot. At 4 months, mice appeared to improve from the initial 4 days of exposure to the task, with a modal unique arm sequence of four, to a unique arm sequence of five in the last 4 days of the task. (E) Plots of the change in the unique sequence scores over each block of testing. The unique sequence score considers both the length and the frequency of the unique sequences. On average, the unique sequence score was 8.70 ± 0.25 (*n* = 58) at 4 months, 8.50 ± 0.31 (*n* = 46) at 8 months, 9.06 ± 0.42 (*n* = 31) at 12 months and 9.22 ± 0.49 (*n* = 22) at 18 months. (F) Distribution of adjacent arm movements. Mice show an inclination to move to the second adjacent arm clockwise or anti‐clockwise from the current arm position (91.44% ± 0.04% mean normalised frequency across all ages). In contrast, mice were disinclined to visit the same arm (12.12% ± 0.02% mean normalised frequency across all ages), or the next adjacent arm (55.64% ± 0.02% mean normalised frequency across all ages). (G) Plots showing daily performance in the nine‐arm radial maze (mean ± SEM; black solid and shaded areas) across the 16 sessions for the decision time at 4, 8, 12 and 18 months of age. On average the mean decision‐making time was 64.06 ± 4.71 s (*n* = 58) at 4 months, 77.14 ± 10.34 s (*n* = 46) at 8 months, 68.91 ± 14.50 s (*n* = 31) at 12 months and 60.44 ± 18.35 s (*n* = 22) at 18 months. Decision‐making time was the only parameter examined that exhibited any significant age‐related change in the coefficient of variability, with an increase from 0.56 at 4 months to 1.42 at 18 months (*R* = 0.9985, *R* ^2^ = 0.9971, *p* < 0.025). (H) Plots showing daily performance in the nine‐arm radial maze (mean ± SEM; black solid and shaded areas) across the 16 sessions for the number of errors observed at 4, 8, 12 and 18 months of age. The population mean error per day was 3.22 ± 0.11 (*n* = 58) at 4 months, 3.33 ± 0.18 (*n* = 46) at 8 months, 3.12 ± 0.24 (*n* = 31) at 12 months and 3.04 ± 0.26 (*n* = 22) at 18 months. Welch's one‐way ANOVA testing showed no significant changes with age for either sequence score (*p* = 0.5505), decision time (*p* = 0.7009) or errors (*p* = 0.8060), with post hoc Dunnett's multiple comparisons between ages showing no significant differences for any of these metrics. (I) Plots of the mean learning rate in the nine‐arm radial maze at each age examined. The mean learning rate was 0.09 ± 0.02 (*n* = 58) at 4 months, 0.11 ± 0.02 (*n* = 46) at 8 months, 0.08 ± 0.02 (*n* = 31) at 12 months and 0.03 ± 0.02 (*n* = 22) at 18 months. Welch's one‐way ANOVA testing demonstrated a significant change with age (*p* = 0.0258), with post hoc Dunnett's multiple comparisons showing only a significant difference between 8 and 18 months of age (*p* = 0.0368).

The nine‐arm radial maze test was chosen so that we could quantify apparent learning strategies in a more complex and cognitively challenging environment. Mice explored most of the nine‐arm radial maze during each test (Figure 2A). The heat map for the cumulative time spent by an individual mouse shows how the center of the platform and the middle of each arm were least explored, reflecting the exposed nature of these locations. The example shown in Figure 2A illustrates a mouse that failed to enter one of the arms on this single trial. The exploratory ratio of the cohort over each 16 days of testing was similar at all ages examined, indicating little change in arm preference (Figure 2B). As well as showing no arm preference, the amount of time spent exploring the arms did not change with age (Figure 2C).

Analysis of unique sequence length in the nine‐arm radial maze demonstrated that an apparent exploration strategy was adopted in four to five arm visits prior to an error by revisiting a previously explored arm. Mice always performed better than chance levels, given that a simple stochastic model resulted in a modal value of two unique arm choices. Therefore, behaviour in the nine‐arm radial maze demonstrates an animal's ability to make choices about which arm to explore, and in what order to explore these arms to minimise visiting a previously explored arm. The unique sequence length consisted of all possible unique sequences during a trial and was not limited to the first observed unique sequence (Figure 2D). At 4 months, when mice were first exposed to the maze, the modal value shifted from an average of four to five unique arm choices in the last 4 days of testing, suggesting an improvement in the apparent strategy adopted by the mice (*n* = 58). At 18 months, mice showed the opposite trend with a modal value of five unique arm choices in the first 4 days, reducing to a modal value of four at the end of the 16‐day session (*n* = 22). This observation could be taken as evidence of an age‐related decline in performance. However, the unique sequence length demonstrated choices above chance levels for mice at the beginning of the 8‐, 12‐ and 18‐month‐old test sessions, suggesting that long‐term learning effects existed for this cohort. Learning effects were also evident in the overall unique sequence score that considers both the length and the frequency of the unique sequence (Figure 2E). The greatest improvement in the USS was apparent at the 4‐ and 8‐month testing periods, but by 12‐ and 18‐months, the score started relatively high at the beginning of each session. This suggests that mice tested at 18 months showed an immediate ability to chain unique arm choices when reintroduced to the nine‐arm radial maze test with little sign of any initial decrease at the beginning of the 16‐day trial period. The age‐related stability in the exploration strategy adopted by these mice was also apparent from the preferred adjacent arm choice (Figure 2F). For a nine‐arm radial maze, we consider there to be −4 to +4 adjacent arm movements with respect to clockwise or anti‐clockwise movement, with entry into the same arm scored as 0. Analysing a total of 16,347 decisions, mice preferred to move −2 or +2 adjacent arms at a normalised frequency of 0.9 ± 0.04 compared to 0.5 ± 0.02 for a move of −1 or +1 and only 0.1 ± 0.02 for an arm re‐entry. Mice were therefore more inclined to visit arms that were not the same arm or immediately adjacent, and this was observed in every age group. We propose that mice adopt a specific exploration strategy at 4 months of age and remember this strategy throughout the testing period.

### 3.5 Learning Effects Are Less Evident at Older Ages, but Performance Is Still High

Decision‐making time in the nine‐arm radial maze at 4, 8 and 12 months of age also showed a non‐monotonic trajectory with a brief increase in the first days of the trial, followed by a more gradual decrease in decision‐making time (Figure 2G). Like the unique sequence score, this trend was less apparent at 18 months where decision time was consistent across the 16 days of the trial. Errors followed a similar trend to sequence score and decision time at 4, 8 and 12 months of age, with a brief increase in error accumulation before a gradual decrease (Figure 2H). Mice showed improved error performance at 18 months, suggesting retained long‐term memory. Like other measures, the average velocity in the nine‐arm radial maze (data not shown) also appeared to drop in the first few days of testing, before increasing during testing. However, once again, Welch's one‐way ANOVA testing showed no significant changes in this parameter with age (*p* = 0.4188). Finally, learning rates in the nine‐arm radial maze also exhibited a characteristic non‐monotonic trajectory with an initial increase in learning rate over the first few days of testing, followed by a gradual decrease across the learning period (Figure 2I). The learning rate did exhibit a significant decrease by 18 months of age that could be interpreted as evidence for cognitive decline. However, error rates, decision‐making time, and unique sequence scores all suggested that performance was maintained at a high level at these older ages.

### 3.6 The Cognitive Performance of Individual Mice Could Be Assessed Across Their Adult Life

**FIGURE 3:**
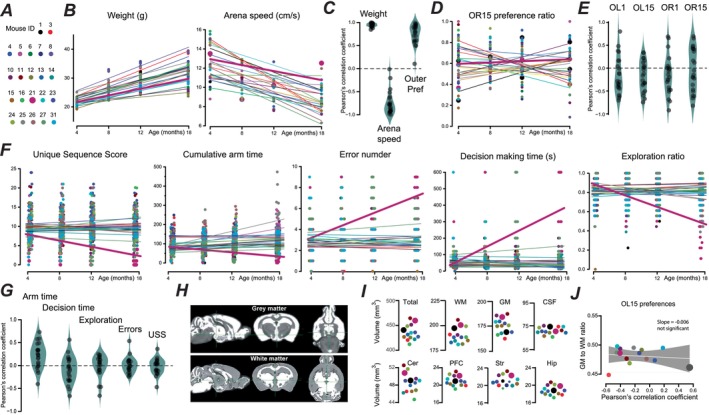
Changes in cognitive ability across the adult lifespan are not correlated with final brain volume. (A) Mouse ID colour code with mouse 21 highlighted with a larger symbol. (B) The left‐hand scatter plot illustrates the weight of individual mice recorded at different ages, with the results of weighted linear regression for each animal. The right‐hand scatter plot illustrates the speed of each animal in the open field test at different ages. (C) Violin plot of the Pearson's correlation coefficients obtained for each animal when analysing weight, arena speed and the proportion of time spent in the outer area of the open field arena. (D) Scatter plot of data from the preference ratios for object recognition tests with a 15‐min retention time (OR15) with the results of weighted linear regression for each animal. (E) Violin plots of the Pearson's correlation coefficients obtained for each animal when analysing the OR1, OR15, OL1 and OL15 preference ratios. (F) Scatter plots illustrating results from the nine‐arm radial maze test when extracting information from unique sequence scores, cumulative arm time, error number, decision‐making time and exploration ratios for all 22 mice. (G) Violin plots of Pearson's correlation coefficient for all mice obtained for five aspects of the nine‐arm radial maze test and the object preference test. (H) representative example of MRI data obtained for a single mouse used in this study. (I) Scatter plots of data extracted from MRI analysis for 14 mice. The average total brain volume was 435.0 ± 4.4 mm^3^ in this cohort, with greater white matter (189.0 ± 2.5 mm^3^) compared to grey matter content (175.3 ± 2.5 mm^3^). There was no significant difference between the brain volume of the left (216.5 ± 7.7 mm^3^) and the right (218.4 ± 8.81 mm^3^) hemispheres. Several cortical and subcortical structures were segmented and quantified. The average prefrontal cortex volume was 21.1 ± 1.2 mm^3^, the hippocampal volume was 18.8 ± 1 mm^3^, the striatal volume was 20.8 ± 1.1 mm^3^ and the cerebellar volume was 48.6 ± 2.1 mm^3^. (J) Scatter plot comparing the ratio of GM to WM in each animal with the Pearson's correlation coefficients obtained from analysis of the object preference ratio changes across the adult ages examined for all 14 mice. The gradient of the line (solid white line) obtained from linear regression analysis (with 95% confidence limits shown with grey shaded area) was consistent with a lack of any relationship between these two parameters as evidenced by a Pearson's correlation coefficient of −0.01 (*p* > 0.05 level, ANOVA).

The main advantage of a longitudinal study is the ability to track changes in an individual across their lifespan. For comparative purposes, we first undertook longitudinal testing for parameters that involved little (if any) cognitive load in the 22 female mice (Figure 3A). Weighted linear regression demonstrated that weight increased in all animals, as evidenced by Pearson's correlation coefficient of at least 0.84, with an average of 0.94 ± 0.04 and a maximum of 0.99 (Figure 3C). Hypothesis testing of the fits for individual animals with one‐way ANOVA resulted in highly significant (*p* < 0.0001) correlations for all mice. Longitudinal analysis of the open field test data demonstrated a strong negative correlation between arena speed and age (Figure 3B) that resulted in significant weighted fits (*p* < 0.05) in just over half of the mice (mouse 1, 3, 8, 10, 11, 12, 14, 15, 16, 23, 24, 25, and 27), with the lowest *p* value observed for mouse 8 (*p* = 0.0004). The Pearson's correlation coefficients for these fits ranged from −0.02 to −0.99 with an arithmetic mean of −0.75 ± 0.05 (Figure 3B). This analysis pipeline was also undertaken for measurements of exploration in the outer region of the arena, where positive correlations were observed for all animals, but a significant fit was only found for mouse 13 (*p* = 0.02). The Pearson's correlation coefficients for these fits ranged from 0.01 to 0.99 with an arithmetic mean of 0.75 ± 0.05 (Figure 3C). Therefore, the open field test data provided clear evidence to support an age‐related decline in movement with age, but the enhanced thigmotaxis that was apparent in this cohort (see Figure 1C) did not reach significance for individual mice.

The short‐term working memory of the 22 mice was assessed using novel object recognition and location testing, with preference ratios calculated for OR1, OR15, OL1 and OL15 over 4 days of testing, making a total of 16 observations for each mouse. We have only shown data obtained for OR15 (see Figure 3D) as this appeared to be the most stable trait in our cohort, with little change in the correlation for any individual animal tested. What is also clear from this analysis is that the age‐related changes in measures such as OR15 lack consistency across the cohort. Similarly, for OL1, the weighted linear regression analysis indicated that none of the 22 mice exhibited any age‐related change in performance, and the Pearson's correlation coefficients ranged from −0.70 to 0.79 with an arithmetic mean of −0.15 ± 0.08. For OL15, Pearson's correlation coefficients ranged from −0.74 to 0.33 with an arithmetic mean of −0.16 ± 0.07. Similarly, no significant fits were observed for OR1 and OR15 data, and the Pearson's correlation coefficients ranged from −0.92 to 0.68 with an arithmetic mean of −0.16 ± 0.09 for OR1 compared to a range of −0.82 to 0.68 with an arithmetic mean of −0.03 ± 0.09 for OR15 (Figure 3E). Therefore, it would appear from this analysis that at an individual level there was little decline in an individual's cognitive performance from 3 to 18 months of age and no consistent change in these parameters within the cohort.

The nine‐arm radial maze test was performed on 64 separate occasions during the life of each mouse, and we have focused on five specific aspects of the nine‐arm radial maze: unique sequence score (USS), error number, decision‐making time, cumulative arm time and exploration ratio (Figure 3F). The USS explored whether mice make choices above chance levels when exploring the maze. Weighted linear regression analysis indicated stability of this trait in most animals, but mice 16 and 21 did exhibit significant changes (*p* = 0.025 and *p* = 0.00015, respectively). There was an age‐related increase in the USS for mouse 16 with a Pearson's correlation coefficient of +0.37, indicating an improvement in this trait, whereas data from mouse 21 was characterized by a negative correlation of −0.46. Overall, the average value for USS was −0.01 ± 0.04, indicating that USS was a stable trait. Cumulative arm time demonstrates the willingness of mice to explore the exposed arms with a significant improvement in this parameter for 10 out of 22 mice, with positive correlations ranging from +0.18 to +0.74. However, an age‐related decrease in this parameter was observed for mouse 21 (*p* = 0.001) with a Pearson's correlation coefficient of −0.39. On average, Pearson's correlation coefficient was +0.22 ± 0.05 for cumulative arm time, indicating that this cohort was overall less anxious and, therefore, more likely to visit the exposed arms at older ages (Figure 3G). The number of errors made during the nine‐arm radial maze was small and remained stable with age, with an average Pearson's correlation coefficient of +0.018 ± 0.04 (Figure 3G). However, mouse 21 showed an increase in errors (*p* < 0.001) with a Pearson's correlation coefficient of +0.54, and mouse 25 also tended to make significantly more errors with a correlation of +0.27 (*p* = 0.03). Decision‐making time was chosen to assess how quickly mice decided to enter the arms, and this parameter generally decreased with age, with an average Pearson's correlation coefficient of −0.08 ± 0.06. However, two mice did exhibit an increase in decision‐making time with age. Once again, mouse 21 showed a significant (*p* < 0.0001) decline in performance with a positive correlation of 0.57, and mouse 8 also significantly increased decision‐making time (*p* = 0.01) with a positive correlation of 0.31. Finally, the exploration ratio gave an indication of how much of the maze each animal explored. Once again, this was remarkably stable apart from mouse 21, which exhibited a marked decline in the exploration ratio over the testing period with a Pearson's correlation coefficient of −0.64 (*p* < 0.0001). Mice 25 and 26 also exhibited a decline in the exploration ratio with similar correlations of −0.45 (*p* = 0.0002). However, the population declined only slightly with an average Pearson's correlation coefficient of just −0.01 ± 0.05 (*n* = 22) as 14 of the mice exhibited small positive correlations with a maximum of +0.27. Mouse 21 was the only animal that exhibited consistent evidence of cognitive decline across the test period, with a reduction in the unique sequence score, an increase in errors when exploring the arms, longer decision‐making times prior to entering the arms, less time spent exploring the arms and a reduced exploration ratio. However, overall, the strength and direction of the correlations for all five parameters extracted from the nine‐arm radial maze data demonstrated less variability (see Figure 3F,G) compared to the correlations extracted from the object recognition/location tests (see Figure 3D,E).

### 3.7 Individual Cognitive Performance Did Not Correlate With Macroscopic Measures of Brain Volume

Following ex vivo structural MRI analysis, total white matter (WM), grey matter (GM) and cerebrospinal fluid (CSF) volumes were quantified in a subset of animals (*N* = 14) that had completed behavioural testing up to 18 months of age (Figure 3H). There was no significant difference between brain volume in the left (216.5 ± 7.7 mm^3^) and the right (218.4 ± 8.81 mm^3^) hemispheres, and WM volumes were greater than GM volumes, with the CSF taking up 16% of the total brain volume. Of note, mouse 21 did not exhibit any unusual brain volumes compared to the other mice, although this mouse exhibited the clearest evidence of age‐related cognitive decline (Figure 3I). Finally, we found no significant relationship between any brain volume measure and the age‐related changes in cognitive abilities. For example, linear regression analysis demonstrates that any age‐related change in object preference estimated did not predict the final grey matter/white matter ratio measured from the MRI volumes in each mouse (Figure 3J).

### 3.8 Early Performance Predicts Cognitive Ability in Later Life

**FIGURE 4:**
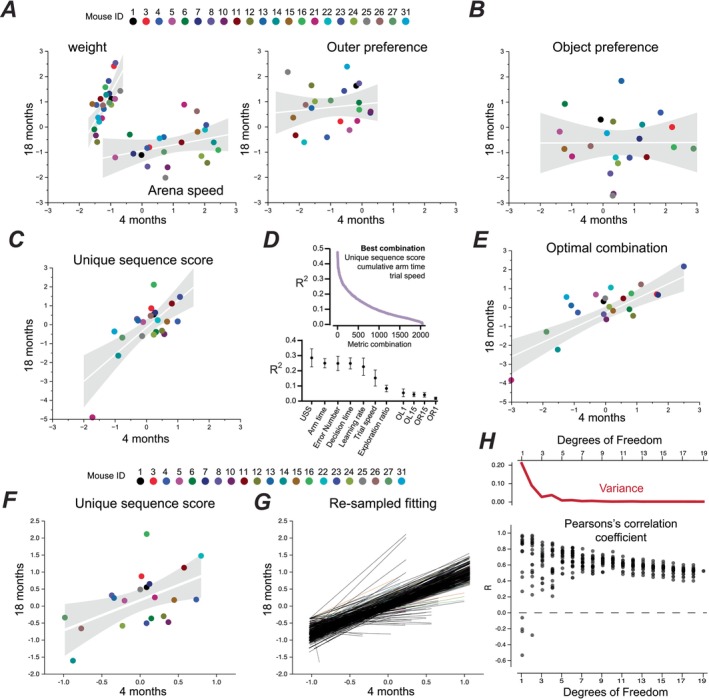
An individual animals performance at 4 months‐of‐age can be a strong predictor of cognitive ability in later life. (A) Scatter plot of the weight, arena speed, and outer arena preference for all 22 mice compared at 4 and 18 months of age using *Z*‐scores to enable comparison between different metrics. The results of weighted linear regression analysis are shown with a solid white line and shaded grey area to illustrate the 95% confidence limits for the fit. (B) Stouffer's *Z*‐score for all measured parameters associated with object preference choice (OL1, OL15, OR1 and OR15) were combined, and scatter plots were constructed to compare performance at different ages. The results of weighted linear regression analysis are shown with a solid white line and shaded grey area to illustrate the 95% confidence limits for the fit. (C) Same conventions as Figure B but for unique sequence score data. (D) Illustration of the linear regression modelling used to determine the predictability of performance metrics. The plots show how metrics were ordered by their mean coefficient of determination (*R* ^2^), and then all possible combinations of metrics were ordered according to their overall best mean *R* ^2^. (E) A scatter plot for the combined metric of unique sequence length, cumulative arm time and velocity at 4 months against performance at 18 months (*R* = 0.7899, *R* ^2^ = 0.6239, *p* < 0.0001, *n* = 22), showing high correlation and levels of predictability. (F) A scatter plot of unique sequence score at 4 and 18 months with mouse 21 removed from the analysis. (G) The black solid lines are the result of linear fitting of 441 re‐sampled data‐sets obtained by randomisation of results from the 21 mice shown in panel F. (H) Scatter plot of the Pearson's correlation coefficients obtained from fits in panel G are plotted according to the degrees of freedom. The solid red trace above the scatter plots shows the reduction in the variance of the data obtained at each degree of freedom.

The main conclusion of the analysis shown in Figure 3 is that many traits associated with cognitive performance are remarkably stable over the adult mouse lifespan. To explore the predictive power of these traits, we explored the relationship between parameters recorded at 4 and 18 months of age using a weighted linear model. As a positive control, an animal's weight at 4 months should be a strong predictor of weight at 18 months because of the strong genetic determinants associated with this trait. As shown in Figure 4A, when we compare the weights of animals at 4 and 18 months, we obtained a Pearson's correlation coefficient of +0.72 that was highly significant (*p* = 0.0002, ANOVA). However, if we look at the speed of the same animals in the open field test or their preference for movement in the outer regions of the arena, we observed little predictive power for these traits (Figure 4A) with Pearson's correlation coefficients of +0.32 (*p* = 0.16) and +0.13 (*p* = 0.56), respectively. This observation implies that these traits are influenced by a variety of internal and external factors that create considerable age‐related variability. Next, we applied the same analysis to the short‐term memory tasks. Once again, the predictive power for these variables was remarkably poor as assessed with our weighted linear model. The correlation coefficient was −0.36 (*p* = 0.06) for OL1, −0.12 (*p* = 0.6) for OL15, +0.01 (*p* = 0.95) for OR1 and −0.15 (*p* = 0.49) for OL15 (data not shown). We combined these four different measures of performance to calculate a Stouffer's combined *Z*‐score for object preference (Figure 4B), but the relationship between these combined metrics also lacked predictive power, with a correlation coefficient of +0.004 (*p* = 0.98, ANOVA). The lack of any correlation between the animal's performance in a short‐term working memory task at 4 and 18 months could reflect the error associated with this measure. Next, we focused on five key metrics produced by the nine‐arm radial maze that rely on longer‐term learning (see Figures 2 and 3). The predictive power of the Unique Sequence Scores obtained at 4 and 18 months (Figure 4C) was as strong as the relationship described for the animal's weight, with a correlation coefficient of +0.72 (*p* = 0.0001). Significant correlations were also observed for decision‐making time and errors, with a Pearson's correlation coefficient of +0.59 (*p* = 0.004) and +0.62 (*p* = 0.002), respectively. However, significance was not observed for the exploration ratio and the cumulative arm time measures described in Figure 3. Therefore, it is clear from this analysis that not all metrics of cognitive performance performed early in life enable predictions to be made about performance later in life.

### 3.9 Some Measures of Performance Are Better Predictors of Future Performance Than Others

To address whether performance metrics can be more efficiently combined to produce a more predictive strategy, the *Z*‐scores for each animal were combined using all possible inclusive and exclusive combinations of metrics (a total of 2047, including single metrics). The highest correlated performance metric combination assessed by the coefficient of determination across all age groups (Figure 4D), was a combination of sequence length, cumulative arm time and velocity (*R* ^2^ = 0.48, *n* = 22). The worst possible combination was decision‐making time, learning rate, velocity, OR1, OR15, OL1 and OL15 (*R* ^2^ < 0.01, *n* = 22). Mice in 1‐ and 15‐min object location tests performed better than the object recognition test, suggesting novel object assessment with novel location measures could be more reliable than novel object assessment without novel location measures (Benice et al. 2006). Plotting individual animals using a combined *Z*‐score of sequence length, cumulative arm time and velocity for 18 versus 4 months performance yields a statistically significant correlation with a Pearson's correlation coefficient of 0.79 (ANOVA, *p* < 0.0001). This strong correlation suggests a high level of predictability from early age performance (Figure 4E). For comparison, the worst combination of decision‐making time, learning rate, velocity, OR1, OR15, OL1 and OL15 yielded a statistically non‐significant correlation (ANOVA, *p* = 0.84) with a very low Pearson's correlation coefficient of just 0.0447 (data not shown). Overall, these results demonstrate that cognitive ability is a stable trait across adult life in C57Bl6 female mice, but consideration needs to be given to the metrics chosen to assay cognitive performance.

For the cohort of 22 female adult mice used in this longitudinal study, a single mouse exhibited signs of cognitive decline, as assessed by their performance in key aspects of the nine‐arm radial maze (see Figure 3). The reasons for this animal's decline in performance are not clear. However, removing this single animal from our final analysis did not alter the predictive power of the observations made at 4 months. For example, the USS plot of performance at 4 and 18 months of age still gave a significant correlation (ANOVA, *p* = 0.015) with a Pearson's correlation coefficient of +0.52 (Figure 4F), and the best combination of metrics still gave a significant correlation (ANOVA, *p* = 0.010) with a Pearson's correlation coefficient of +0.55. To explore other potential sources of bias within our interpretation of these results, a cross‐validation approach was undertaken that involved re‐sampling of the data prior to linear model analysis (Figure 4G). Pearson's correlation coefficients were obtained from the 441 linear fits, and the variance for these measures of correlation was then plotted at all degrees of freedom (Figure 4H). Considerable variability in the correlation coefficient was apparent when the degrees of freedom included in the fit were low (< 10), but the variance of this coefficient was remarkably stable when the degrees of freedom were high. Therefore, the longitudinal testing approach undertaken in this study has enabled a single measure of performance to robustly predict cognitive performance later in life even when relatively few independent observations (> 10) were included in the sample set.

## 4 Discussion

Human longitudinal studies have identified many risk factors for dementia, but what has become clear from these studies is that individuals who perform better in early life are more likely to be performing well in older age (Deary et al. 2013; McCall 1977). Our longitudinal testing of female C57Bl/6J mice has demonstrated that cognitive ability, at least on the specific tasks tested in this study, is a stable trait in mice. It was also notable that, overall, our study did not reveal any significant cognitive decline in female mice up to 18 months of age. To analyse changes in cognitive ability, we combined weighted *z*‐scores from a variety of cognitive tests and performed linear regression analysis on data obtained at 4, 8, 12 and 18 months of age. The correlation coefficients obtained from this analysis demonstrated that a significant proportion of the variability in cognitive performance observed at 18 months of age could be explained by variability observed at 4 months of age, and this correlation could be improved further when the analysis was restricted to the three most stable traits (see Figure 4D). Our analysis demonstrates how some performance metrics are better predictors of performance in later life than others. For example, speed in the open field arena and performance in short‐term memory tasks have little obvious predictive power. In contrast, the most robust indicator of cognitive performance in later life (18 months) was the unique sequence score at 4 months of age. This metric captures an animal's ability to systematically explore a 9‐arm radial maze with a high frequency of unique arm visits that avoid returning to unexplored regions of the maze. We speculate that measures of performance that require a greater cognitive load could offer greater predictive power in a longitudinal study of this type.

Stability of cognitive ability has been demonstrated in other recent longitudinal testing studies in mice, albeit over shorter time periods than our study. For example, one longitudinal study in female C57Bl/6J mice examined exploration during the oestrous cycle and reached a similar conclusion for individual mice over a few weeks of testing (Levy et al. 2023). Another study using a fully automated home cage monitoring system demonstrated stability of cognitive ability in wild‐type mice over several months of testing, and a study of short‐term memory in an APP/PS1 mouse model of AD reported little cognitive decline between 1 month and 8 months of age (Soto et al. 2023). When monitored over 12 months, free social interactions in female mice were also shown to be stable in the wild‐type control strain used in a mouse model of AD, the 5xFAD strain (Kosel et al. 2019). The accuracy of the older mice that we describe at 18 months of age could be attributed to learning effects associated with repeated exposure to the cognitive test. Alternatively, elevated anxiety or lack of motivation in older naïve mice could explain the poor performance of older cohorts studied in more conventional cross‐sectional studies. However, we would argue that learning effects do not mask age‐related decline in performance but should be considered a key component of the cognitive reserve that we and others have now observed in longitudinal studies of mice undertaken over the adult lifespan.

Early life experience and epigenetic changes during prenatal and early postnatal periods of development are likely to be key factors in producing the cognitive reserve necessary for healthy ageing. Moreover, the considerable inter‐animal variability we observed in our measures of cognitive performance is probably influenced by these factors. Understanding the contribution of early life events to the generation of a healthy adult brain is one area of research that should benefit from the adoption of longitudinal testing approaches of the type described in this study. The electrophysiological data we report add further support to our earlier suggestion that changes in neuronal excitability could help maintain cognitive ability in later life (Lucaci et al. 2023). Changes in the magnitude of a slow AHP observed following repetitive AP firing have been reported in layer 5 neurons of the monkey PFC with age, but these alterations were not associated with cognitive performance. Studies of poor cognitive performance in aged rodents have reported links to the AHP in pyramidal neurons of the hippocampus, but studies of this type have focused on the post‐burst AHP. Therefore, the significance of the AHP changes we observe following single APs needs to be examined further and extended to burst‐firing‐induced AHPs. Nevertheless, our results support the established view that changes in neuronal excitability take place during ageing. In contrast, our anatomical observations failed to identify brain volume differences associated with better cognitive performance, as measured by ex vivo structural MRI. Previous longitudinal functional MR imaging of the mouse brain has reported changes in the default mode network (Carli et al. 2023; Vasilkovska et al. 2023) with an overall reduction in (resting state) functional connectivity as determined from BOLD signals in both wild‐type and disease models. Changes in (resting state) functional connectivity are often considered responsible for the maintenance of cognitive ability and individual differences in the resting state BOLD signal observed in the DMN have been correlated with improved cognitive ability in human studies (Wang et al. 2021). However, these human neuroimaging studies cannot provide insights into the cellular, molecular and/or anatomical changes in the brain that should be responsible for differences in functional connectivity. Our current study was limited to ex vivo structural imaging at the completion of longitudinal testing. Thus, longitudinal in vivo studies in mice are needed to identify the early life events and associated biomarkers that may help establish the cognitive reserve necessary for healthy ageing, including exploration of functional connectivity changes in the DMN of mice. Such studies also provide an opportunity to link these data to molecular, cellular and physiological measurements in the same animal.

One important advantage of longitudinal testing is the reduction in sample size required to achieve confidence in an observation. For example, cross‐sectional studies in humans that attempted to correlate years in education with fluid intelligence required data from half a million individuals and resulted in a correlation coefficient of just 0.3 (Tari et al. 2023). In contrast, a Canadian study on World War II veterans was one of the earliest longitudinal studies to demonstrate the predictive power of educational attainment with a far smaller dataset. For 260 participants aged 25 at the age of recruitment, it was found that years in education were the best predictor of their cognitive abilities at age 65, with a correlation of 0.8 (Schwartzman et al. 1987). Similarly, the Betula prospective cohort study in Sweden used data from 262 World War II recruits at age 18 to demonstrate that general cognitive abilities were extremely stable up to 65 years of age, with a correlation coefficient of 0.9 (Rönnlund et al. 2015). A correlation coefficient of 0.7 was also calculated in a longitudinal study performed in a UK study that tracked cognitive ability in a cohort of just 106 individuals between the ages of 11 and 90 (Deary et al. 2013). A meta‐analysis of longitudinal studies performed since the 1920s has led to the conclusion that cognitive performance from as early as 7 years of age can be an accurate predictor of healthy ageing (Breit et al. 2024). In our study, we were able to identify strong predictors of future performance based upon a sample size of just 22 mice. Indeed, the results of our re‐sampled fitting demonstrated how tolerant a performance measure such as the USS is to low sample numbers. By harnessing the predictive power of longitudinal testing, future studies of the type described in our work should help better identify the key biological and environmental factors that enable healthy brain ageing. More generally, this longitudinal study highlights the importance of understanding how a healthy brain is constructed during early life, and future studies of this type will help identify the mechanisms that generate cognitive reserve and protect against dementia in later life.

## Author Contributions

R.A. contributed to experimental design, performed all longitudinal testing experiments and analysed data. G.M. contributed to data analysis. D.D. and F.Z. performed electrophysiological experiments. A.E. and P.C. contributed to experimental design. D.C. and E.K. performed MRI data collection. A.C.V. contributed to data interpretation. C.E. contributed to data analysis. P.C. designed the experiments and wrote the paper. S.G.B. designed the experiments, analysed data and wrote the paper.

## Conflicts of Interest

The authors declare no conflicts of interest.

## Data Availability

The data that support the findings of this study are available on request from the corresponding author. The data are not publicly available due to privacy or ethical restrictions.

## References

[acel70287-bib-0001] Abuhamdah, R. M. , M. D. Hussain , P. L. Chazot , and A. Ennaceur . 2016. “Pre‐Training in a Radial Arm Maze Abolished Anxiety and Impaired Habituation in C57BL6/J Mice Treated With Dizocilpine.” Physiology & Behavior 164: 353–360. 10.1016/j.physbeh.2016.06.017. 27317838

[acel70287-bib-0031] Benice, T. S. , A. Rizk , S. Kohama , T. Pfankuch , and J. Raber . 2006. “Sex‐differences in Age‐related Cognitive Decline in C57BL/6J Mice Associated with Increased Brain Microtubule‐associated Protein 2 and Synaptophysin Immunoreactivity.” Neuroscience 137, no. 2: 413–423. 10.1016/j.neuroscience.2005.08.029. 16330151

[acel70287-bib-0002] Breit, M. , V. Scherrer , E. M. Tucker‐Drob , and F. Preckel . 2024. “The Stability of Cognitive Abilities: A Meta‐Analytic Review of Longitudinal Studies.” Psychological Bulletin 150: 399–439. 10.1037/bul0000425. 38330347 PMC11626988

[acel70287-bib-0003] Brito, D. V. C. , F. Esteves , A. T. Rajado , et al. 2023. “Assessing Cognitive Decline in the Aging Brain: Lessons From Rodent and Human Studies.” NPJ Aging 9: 23. 10.1038/s41514-023-00120-6. 37857723 PMC10587123

[acel70287-bib-0004] Carli, S. , L. Chaabane , G. de Rocco , et al. 2023. “A Comprehensive Longitudinal Study of Magnetic Resonance Imaging Identifies Novel Features of the Mecp2 Deficient Mouse Brain.” Neurobiology of Disease 180: 106083. 10.1016/j.nbd.2023.106083. 36931532

[acel70287-bib-0005] Deary, I. J. , A. Pattie , and J. M. Starr . 2013. “The Stability of Intelligence From Age 11 to Age 90 Years: The Lothian Birth Cohort of 1921.” Psychological Science 24: 2361–2368. 10.1177/0956797613486487. 24084038

[acel70287-bib-0006] Guilloux, J. P. , M. Seney , N. Edgar , and E. Sibille . 2011. “Integrated Behavioral z‐Scoring Increases the Sensitivity and Reliability of Behavioral Phenotyping in Mice: Relevance to Emotionality and Sex.” Journal of Neuroscience Methods 197: 21–31. 10.1016/j.jneumeth.2011.01.019. 21277897 PMC3086134

[acel70287-bib-0030] Jenkinson, M. , C. F. Beckmann , T. E. J. Behrens , M. W. Woolrich , and S. M. Smith . 2012. “FSL.” NeuroImage 62, no. 2: 782–790. 10.1016/j.neuroimage.2011.09.015. 21979382

[acel70287-bib-0007] Kang, Y. K. , B. Min , J. Eom , and J. S. Park . 2022. “Different Phases of Aging in Mouse Old Skeletal Muscle.” Aging 14: 143–160. 10.18632/aging.203812. 35017317 PMC8791220

[acel70287-bib-0008] Katz, R. J. , K. A. Roth , and B. J. Carroll . 1981. “Acute and Chronic Stress Effects on Open Field Activity in the Rat: Implications for a Model of Depression.” Neuroscience and Biobehavioral Reviews 5: 247–251. 10.1016/0149-7634(81)90005-1. 7196554

[acel70287-bib-0009] Kosel, F. , P. Torres Munoz , J. R. Yang , A. A. Wong , and T. B. Franklin . 2019. “Age‐Related Changes in Social Behaviours in the 5xFAD Mouse Model of Alzheimer's Disease.” Behavioural Brain Research 362: 160–172. 10.1016/j.bbr.2019.01.029. 30659846

[acel70287-bib-0010] Levy, D. R. , N. Hunter , S. Lin , et al. 2023. “Mouse Spontaneous Behavior Reflects Individual Variation Rather Than Estrous State.” Current Biology 33: 1358–1364. 10.1016/j.cub.2023.02.035. 36889318 PMC10090034

[acel70287-bib-0011] Lipnicki, D. M. , S. R. Makkar , J. D. Crawford , et al. 2019. “Determinants of Cognitive Performance and Decline in 20 Diverse Ethno‐Regional Groups: A COSMIC Collaboration Cohort Study.” PLoS Medicine 16: e1002853. 10.1371/journal.pmed.1002853. 31335910 PMC6650056

[acel70287-bib-0012] Livingston, G. , J. Huntley , K. Y. Liu , et al. 2024. “Dementia Prevention, Intervention, and Care: 2024 Report of the Lancet Standing Commission.” Lancet 404: 572–628. 10.1016/S0140-6736(24)01296-0. 39096926

[acel70287-bib-0013] Lucaci, D. , X. Yu , P. Chadderton , W. Wisden , and S. G. Brickley . 2023. “Histamine Release in the Prefrontal Cortex Excites Fast‐Spiking Interneurons While GABA Released From the Same Axons Inhibits Pyramidal Cells.” Journal of Neuroscience 43: 187–198. 10.1523/JNEUROSCI.0936-22.2022. 36639899 PMC9838703

[acel70287-bib-0014] McCall, R. B. 1977. “Childhood IQ'S as Predictors of Adult Educational and Occupational Status.” Science 197: 482–483. 10.1126/science.197.4302.482. 17783247

[acel70287-bib-0015] Moore, T. L. , M. Medalla , S. Ibañez , et al. 2023. “Neuronal Properties of Pyramidal Cells in Lateral Prefrontal Cortex of the Aging Rhesus Monkey Brain Are Associated With Performance Deficits on Spatial Working Memory but Not Executive Function.” Geroscience 45: 1317–1342. 10.1007/s11357-023-00798-2. 37106282 PMC10400510

[acel70287-bib-0016] Perlman, R. L. 2016. “Mouse Models of Human Disease: An Evolutionary Perspective.” Evolution, Medicine, & Public Health 2016: 170–176. 10.1093/emph/eow014. 27121451 PMC4875775

[acel70287-bib-0017] Prince, M. , R. Bryce , E. Albanese , A. Wimo , W. Ribeiro , and C. P. Ferri . 2013. “The Global Prevalence of Dementia: A Systematic Review and Metaanalysis.” Alzheimer's & Dementia 9: 63–75.e2. 10.1016/j.jalz.2012.11.007. 23305823

[acel70287-bib-0018] Rönnlund, M. , A. Sundström , and L. G. Nilsson . 2015. “Interindividual Differences in General Cognitive Ability From Age 18 to Age 65 Years Are Extremely Stable and Strongly Associated With Working Memory Capacity.” Intelligence 53: 59–64. 10.1016/j.intell.2015.08.011.

[acel70287-bib-0019] Schwartzman, A. E. , D. Gold , D. Andres , T. Y. Arbuckle , and J. Chaikelson . 1987. “Stability of Intelligence: A 40‐Year Follow‐Up.” Canadian Journal of Psychology 41: 244–256. 10.1037/h0084155. 3502899

[acel70287-bib-0020] Shoji, H. , K. Takao , S. Hattori , and T. Miyakawa . 2016. “Age‐Related Changes in Behavior in C57BL/6J Mice From Young Adulthood to Middle Age.” Molecular Brain 9: 1–18. 10.1186/s13041-016-0191-9. 26822304 PMC4730600

[acel70287-bib-0021] Soto, P. L. , M. E. Young , G. M. DiMarco , et al. 2023. “Longitudinal Assessment of Cognitive Function in the APPswe/PS1dE9 Mouse Model of Alzheimer's‐Related Beta‐Amyloidosis.” Neurobiology of Aging 128: 85–99. 10.1016/j.neurobiolaging.2023.03.010. 37120419 PMC10239324

[acel70287-bib-0022] Tari, B. , M. Kunzi , C. P. Pflanz , V. Raymont , and S. Bauermeister . 2023. “Education Is Power: Preserving Cognition in the UK Biobank.” Frontiers in Public Health 11: 1244306. 10.3389/fpubh.2023.1244306. 37841724 PMC10568007

[acel70287-bib-0023] Vasilkovska, T. , M. H. Adhikari , J. van Audekerke , et al. 2023. “Resting‐State fMRI Reveals Longitudinal Alterations in Brain Network Connectivity in the zQ175DN Mouse Model of Huntington's Disease.” Neurobiology of Disease 181: 106095. 10.1016/j.nbd.2023.106095. 36963694

[acel70287-bib-0024] Walker, S. E. , T. C. Wood , D. Cash , M. Mesquita , S. C. R. Williams , and C. Sandi . 2018. “Alterations in Brain Microstructure in Rats That Develop Abnormal Aggression Following Peripubertal Stress.” European Journal of Neuroscience 48: 1818–1832. 10.1111/ejn.14061. 29961949

[acel70287-bib-0025] Wang, R. , M. Liu , X. Cheng , Y. Wu , A. Hildebrandt , and C. Zhou . 2021. “Segregation, Integration, and Balance of Large‐Scale Resting Brain Networks Configure Different Cognitive Abilities.” Proceedings of the National Academy of Sciences of the United States of America 118: 1–11. 10.1073/pnas.2022288118. PMC8201916 34074762

[acel70287-bib-0026] Weber, M. , T. Wu , J. E. Hanson , et al. 2015. “Cognitive Deficits, Changes in Synaptic Function, and Brain Pathology in a Mouse Model of Normal Aging.” eNeuro 2: 1–26. 10.1523/ENEURO.0047-15.2015. PMC4606159 26473169

[acel70287-bib-0027] Whitehead, J. C. , B. A. Hildebrand , M. Sun , et al. 2014. “A Clinical Frailty Index in Aging Mice: Comparisons With Frailty Index Data in Humans.” Journals of Gerontology. Series A, Biological Sciences and Medical Sciences 69: 621–632. 10.1093/gerona/glt136. 24051346 PMC4022099

[acel70287-bib-0028] Wittenberg, R. , M. Knapp , B. Hu , et al. 2019. “The Costs of Dementia in England.” International Journal of Geriatric Psychiatry 34: 1095–1103. 10.1002/gps.5113. 30950106 PMC6618309

[acel70287-bib-0029] Zhang, X. , E. Yacoub , and X. Hu . 2001. “New Strategy for Reconstructing Partial‐Fourier Imaging Data in Functional MRI.” Magnetic Resonance in Medicine 46: 1045–1048. 10.1002/mrm.1296. 11675662

